# Gastric hepatoid adenocarcinoma resulting in a spontaneous gastric perforation: a case report and review of the literature

**DOI:** 10.1186/s12885-017-3357-7

**Published:** 2017-05-25

**Authors:** Junichi Yoshizawa, Satoshi Ishizone, Meguru Ikeyama, Jun Nakayama

**Affiliations:** 1 Department of Surgery, North Alps Medical Center Azumi Hospital, 3207-1 Ikeda, Ikeda-machi, Kitaazumi-gun, Nagano Prefecture, 399-8695 Japan; 2 Department of Molecular Pathology, Shinshu University Graduate School of Medicine, 3-1-1 Asahi, Matsumoto, Nagano Prefecture, 390-8621 Japan; 3 Present Address: Suwa Red Cross Hospital, 5-11-50 Kogandori, Suwa-shi, Nagano Prefecture, 392-8510 Japan

**Keywords:** Alpha-fetoprotein, Case report, Gastric cancer, Gastric perforation, Hepatoid adenocarcinoma

## Abstract

**Background:**

Gastric hepatoid adenocarcinoma (GHAC) is an atypical form of gastric cancer (GC) that has similar tissue morphology to hepatocellular carcinoma and frequently produces alpha-fetoprotein. We present an exceedingly rare case of GHAC resulting in a spontaneous gastric perforation.

**Case presentation:**

A 61-year-old man presented at our institution complaining of abdominal and back pain. A computed tomography scan revealed a spontaneous gastric perforation with a solitary liver tumor and lymph node swelling. Following a diagnosis of advanced-stage GC with a gastric perforation, perforative peritonitis, multiple lymph node metastases, and a solitary metastasis of the lateral segment of the liver, the patient underwent distal gastrectomy. Histopathology of the resected specimen revealed that the tumor cells were arranged in a hepatoid pattern. On immunohistochemical staining, the tumor cells were positive for alpha-fetoprotein and Sal-like protein 4. Thus, the patient was diagnosed with GHAC. Hepatic resection of the solitary liver metastasis was performed. However, recurrence occurred and the patient achieved complete response following tegafur/gimeracil/oteracil-based chemotherapy.

**Conclusions:**

GHAC is a highly malignant histological subtype of GC. We reported on an extremely rare case of GHAC resulting in a spontaneous gastric perforation and reviewed the literature, including epidemiological data, treatment regimens, and the association between GHAC and alpha-fetoprotein-producing GC.

## Background

Hepatoid adenocarcinoma is a malignant cancer manifesting outside the liver that most frequently arises in the stomach, with gastric hepatoid adenocarcinoma (GHAC) accounting for 63% of cases. Hepatoid adenocarcinoma also arises in the ovaries (10%), lungs (5%), bladder (4%), pancreas (4%), and uterus (4%) [1]. GHAC is a rare form of gastric cancer (GC) that accounts for ≤1% of all GCs [2–4]. GHAC has been recognized in approximately 500 cases to date, mainly in case reports and clinical or pathological analyses that were identified from literature searches of the PubMed database using the search terms: “hepatoid adenocarcinoma of the stomach” AND “gastric hepatoid adenocarcinoma” [4, 5]. Among the different GC subtypes, GHAC has comparable histology and functionality to stem cell differentiation, it has pathologically similar tissue morphology to hepatocellular carcinoma (HCC), and it frequently expresses alpha-fetoprotein (AFP) on immunohistochemistry [6, 7]. GHACs progress rapidly, with the majority of patients presenting with lymph node (LN) or liver metastases. The risk of recurrence in GHAC patients is high, even after radical resection. Currently, no standard chemotherapy regimen has been established [8]. For these reasons, the prognosis of patients with GHAC remains especially poor. Liu et al. [2] reported that the 1-, 3-, and 5-year survival rates of GHAC versus non-GHAC patients were 30%, 13%, and 9%, and 96%, 61%, and 44%, respectively. GHAC patients had a statistically significant poorer prognosis than non-GHAC patients [2]. GHAC has no specific symptoms with many common symptoms of GC having been observed (e.g., general fatigue, reduced appetite, gastric distention, epigastric pain, anemia, and melena) [8]. We present an exceedingly rare case of GHAC resulting in a spontaneous gastric perforation and review the literature, including epidemiological data, treatment regimens, and the association between GHAC and AFP-producing GC.

## Case presentation

A 61-year-old man experienced upper abdominal and lower left back pain 1 month and 1 week prior to examination, respectively. He was referred to our hospital after the pain had worsened. The patient experienced spontaneous lumbar and epigastric pain with muscular defense of the epigastrium and accompanying tenderness. Blood test results indicated a white blood cell count of 12,430 /μL, a C-reactive protein level of 0.6 mg/dL, and mild but increasing inflammation. No abnormal findings were reported from the other blood counts, biochemical examinations, and coagulation tests.

**Fig. 1:**
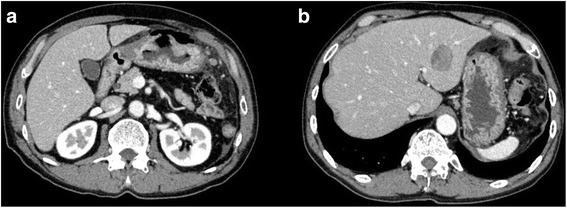
Imaging findings. Abdominal contrast-enhanced computed tomography revealed disruption and thickening of the anterior wall of the gastric antrum with free air (**a**) and a low enhanced lesion (30 mm in diameter) on the lateral segment of the liver (**b**)

**Fig. 2:**
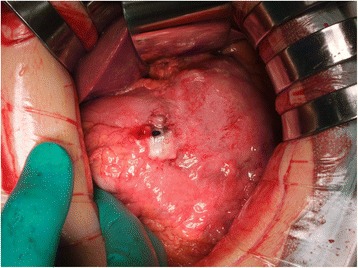
Intraoperative findings. A 7-mm perforation of the anterior wall of the gastric antrum with serous exposure of bulky tumors and opacity ascites

Abdominal contrast-enhanced computed tomography (CT) revealed disruption and thickening of the anterior wall of the gastric antrum. CT also revealed the presence of ascites and free air at the ventral side of the stomach and on the surface of the liver (Fig. 1a). Several enlarged LNs (maximum diameter, 30 mm) were identified along the greater gastric curve and a low enhanced lesion (measuring 30 × 25 mm) was detected on the lateral segment of the liver (Fig. 1b). A diagnosis of advanced-stage GC resulting in a spontaneous gastric perforation, with perforative peritonitis, multiple LN metastases, and a solitary liver metastasis was made and an emergency laparotomy was performed on the same day. A moderate amount of turbid ascites was observed in the abdominal cavity during laparotomy. A 7-mm perforation of the gastric antrum was detected, along with marked thickening of the gastric wall and coarse neoplastic tumors that were attached to the gastric wall (Fig. 2). The tumors were exposed on the serosal surface along the gastric perforation. The patient was diagnosed with advanced-stage GC resulting in a spontaneous gastric perforation. No peritoneal dissemination was observed. Preoperative CT also revealed the presence of metastases in several enlarged LNs along the greater gastric curve, as well as, a number of hardened regions in the lateral segment of the liver. A distal gastrectomy with radical lymphadenectomy and cholecystectomy was performed. Reconstructive surgery was achieved using Billroth II anastomosis. No postoperative complications occurred and the patient was discharged.

**Fig. 3:**
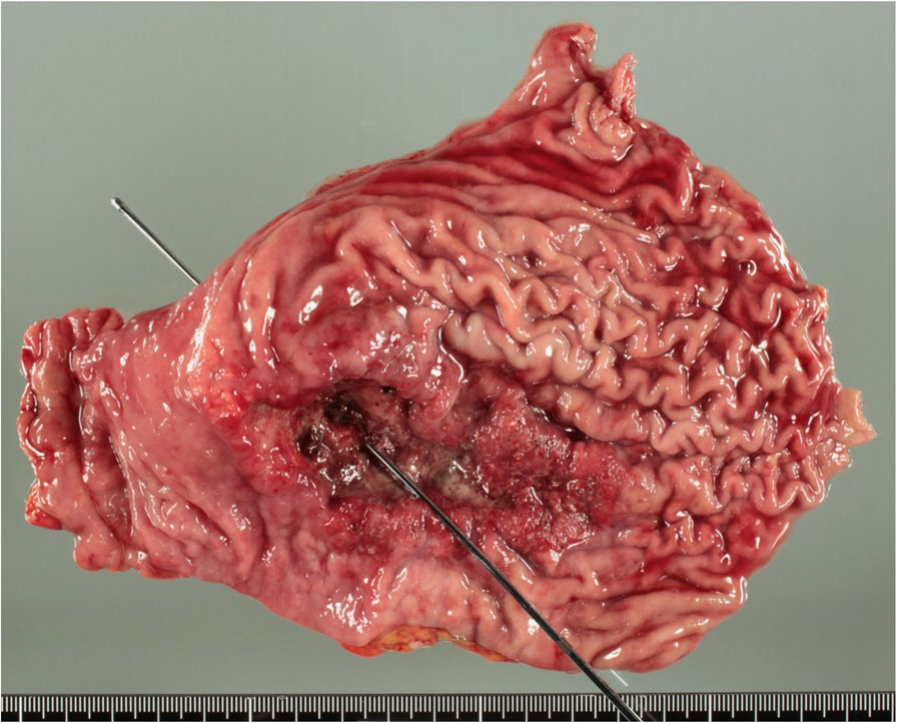
Gross findings. Ulcerative and infiltrative (type 3) tumor of the gastric antrum (approximately 100 × 50 mm) with an infiltrative serosal surface and central perforation of the gastric cancer

**Fig. 4:**
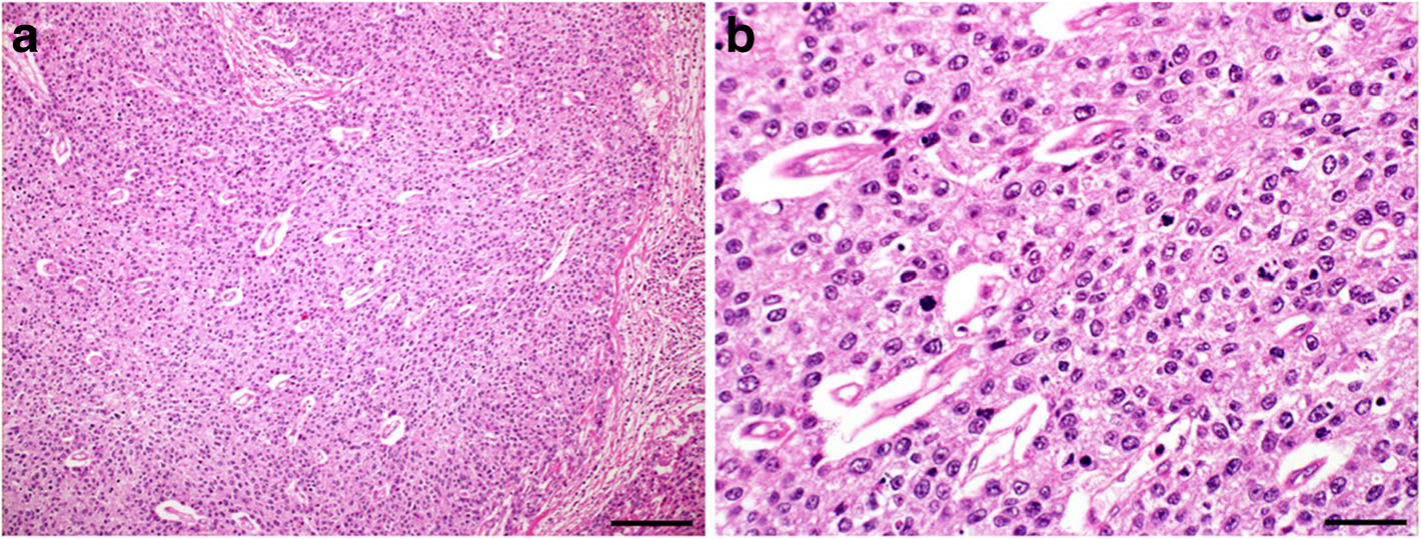
Histopathological findings. Hematoxylin and eosin staining of proliferating tumor cells with solid or thick-trabecular patterns mimicking hepatocellular carcinoma. The scale bars in (**a**) and (**b**) indicate 200 μm and 50 μm, respectively

Gross findings of the resected specimen included an ulcerative and infiltrative (type 3) tumor (approximately 100 × 50 mm) with an infiltrative serosal surface and 7-mm puncture sites (Fig. 3). Microscopic findings revealed that the tumor was comprised of a homogeneous proliferation of polygonal tumor cells with abundant, eosinophilic, and clear cytoplasm. Numerous mitoses were also detected. The tumor cells exhibited solid or thick-trabecular patterns with scanty stroma containing blood vessels that resembled HCC and expansive invasion into the gastric wall (Fig. 4). Features of enteroblastic differentiation and Schiller-Duval bodies were absent.

**Fig. 5:**
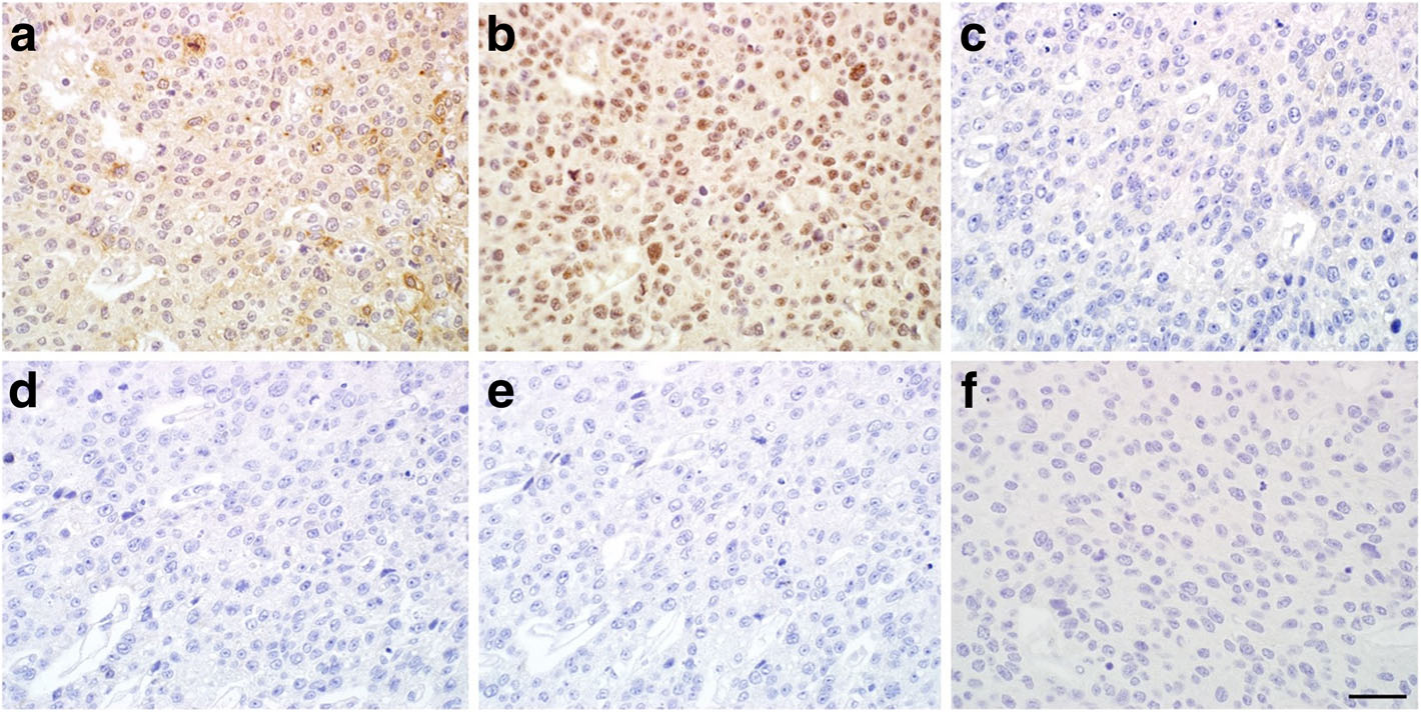
Immunohistochemical analysis. Tumor cells stained positive for (**a**) alpha-fetoprotein and (**b**) Sal-like protein 4, but were negative for (**c**) carcinoembryonic antigen, (**d**) synaptophysin, (**e**) chromogranin A, and (**f**) neural cell adhesion molecule. The scale bar indicates 50 μm

Immunohistochemical staining was performed to characterize the tumor cells (Table 1). The tumor cells stained positive for AFP and Sal-like protein 4 (SALL4), but were negative for carcinoembryonic antigen, synaptophysin, chromogranin A, and neural cell adhesion molecule (Fig. 5a–f). According to these findings, the patient was diagnosed with GHAC. No tumor cells were observed during the cytological examination of ascites. LN metastases were detected in 6 LNs (20%) and a solitary metastasis was detected on the lateral segment of the liver. Subsequently, the patient was classified as having a Stage IV (T4aN2M1) GHAC. Moderate venous and lymphatic infiltration was observed. Postoperative blood biochemical analysis revealed an elevated AFP level (487.4 ng/mL), which supported a diagnosis of GHAC.

**Table 1:** Antibodies used for immunohistochemical analysis

Antibody	Clone	Supplier	Supplier location	Detection kit	Supplier
Carcinoembryonic antigen (CEA)	TF3H8–1 (mouse, monoclonal)	Ventana Medical Systems	Tucson, AZ, USA	Ventana ultraView DAB	Ventana Medical Systems
Synaptophysin	MRQ-40 (rabbit, monoclonal)	Ventana Medical Systems	Tucson, AZ, USA	Ventana ultraView DAB	Ventana Medical Systems
Chromogranin A	LK2H10 (mouse, monoclonal)	Ventana Medical Systems	Tucson, AZ, USA	Ventana ultraView DAB	Ventana Medical Systems
Neural cell adhesion molecule (NCAM)	123C3 (mouse, monoclonal)	Monosan	Uden, The Netherlands	Histofine Simple Stain MAX-PO(M)	Nichirei Biosciences
Alpha-fetoprotein	A0008 (rabbit, polyclonal)	DAKO	Glostrup, Denmark	Histofine Simple Stain MAX-PO(R)	Nichirei Biosciences
Sal-like protein 4 (SALL4)	6E3 (mouse monoclonal)	Abcam	Cambridge, UK	Histofine Simple Stain MAX-PO(M)	Nichirei Biosciences

The patient was followed-up for 11 weeks post-gastrectomy to monitor the occurrence of new LN or liver metastases. Because no new lesions were detected, resection of the lateral segment of the liver was performed, at another hospital, 13 weeks after the initial operation. The patient was confirmed from histopathological examinations as having GHAC with a solitary liver metastasis. An elevated serum AFP level (1214.9 ng/mL) was recorded 6 weeks after hepatic resection and recurrence of LN metastasis was detected on CT. Combination chemotherapy with tegafur/gimeracil/oteracil (S-1) was administered at a dose of 120 mg/day for 2 weeks with a 1-week rest. Three months later, serum AFP levels had normalized and shrinkage of the resected LN was observed. Elevated serum AFP levels were not detected 15 months post-recurrence and complete response of the LN metastasis was achieved.

## Discussion and conclusions

Boureille et al. [9] first reported on the use of elevated serum AFP levels as a biomarker for GC in 1970. In 1977, Okita et al. [10] verified the expression of AFP in GC, through immunohistochemistry, and established the concept of “AFP-producing GC”. AFP-producing GC does not reflect a diagnosis of a specific histological subtype, but rather describes a group of tumor histologies that have the capacity to produce AFP. These include gastric hepatoid, enteroblastic, and yolk sac tumors [11, 12]. It should be noted that GC with enteroblastic differentiation is distinguishable from gastroblastoma, which is a neoplasm that is rarely observed in children or young adults and is considered a low-grade malignancy [13, 14].

In 1985, Ishikura et al. [6] introduced the concept of GHAC after conducting an investigation of AFP-producing GC cases with morphological features mimicking HCC. However, since a proportion of GHAC patients do not express AFP, Nagai et al. [5] suggested that GHAC should be diagnosed based on its histological characteristics, irrespective of its capacity to produce AFP. Since AFP production has been observed in the fetal liver, HCCs, and GHACs, GHAC is considered to represent a gastric carcinoma with hepatic differentiation and morphological similarity to hepatic cells [15].

Inagawa et al. [8] investigated 85 GHAC patients (mean age, 63.5 [range, 44–87] years with a male-to-female ratio of 2.3:1) and reported that GHAC had occurred relatively more frequently in middle-aged men than in elderly men. GHAC originated in the gastric antrum in 60% of patients. Only 13% of patients were diagnosed with early-stage GHAC. Gross findings suggested the presence of type 2 or type 3 ulcerative lesions in 29 (62%) of 47 cases. The majority of patients presented with LN and liver metastases. These findings were consistent with the findings of our case.

GHAC is not associated with specific symptoms and many common symptoms of GC are observed (e.g., general fatigue, reduced appetite, gastric distention, epigastric pain, anemia, and melena) [8]. In our case, the patient developed upper abdominal and back pain, and a spontaneous gastric perforation was detected on CT.

Histopathologically, GHAC resembles HCC. The tumor cells grow, proliferate, and invade surrounding tissues with significant accompanying venous infiltration [11, 16]. GHACs are frequently associated with highly differentiated papillary adenocarcinomas. It is thought that GHACs may arise from these tumors through hepatic differentiation [15–17]. However, such a differentiated adenocarcinoma was not detected in the present case.

AFP and glypican-3 are oncofetal proteins that are produced by the fetal liver, yolk sac tumors, hepatoblastomas, and HCCs [18]. Because these proteins are also frequently expressed in enteroblastic gastric tumors, as well as GHACs, each tumor is classified as GC exhibiting fetal differentiation. SALL4 expression has been observed in the neofetal stomach, primitive germ cell tumors, enteroblastic adenocarcinomas, yolk sac tumors, and GHACs [19]. Since AFP expression is often negative in GHACs, whereas glypican-3 and SALL4 expression are usually positive, glypican-3 and SALL4 are considered potentially more useful clinical biomarkers of GHAC than AFP. Moreover, SALL4 is not expressed in normal liver tissue or HCCs. Therefore, SALL4 expression may be useful for distinguishing GHACs from HCCs [5, 18–20]. In our case, both AFP and SALL4 expression, as well as, morphological features mimicking HCC meant we were able to diagnose the tumor as a GHAC.

Although GC resulting in a gastric perforation is rare (accounting for <1% of all GC cases [21–24]), the possibility of GC should be considered when a diagnosis of a gastric perforation is confirmed. The diagnosis of a gastric perforation in GC patients, either before or during surgery, is not necessarily straightforward, with diagnoses frequently made based on ulcer size, the extent of sclerosis, the presence of LN and liver metastases, and gastric dissemination. In our case, coarse neoplastic tumors and serosal invasion were observed, along with suspected LN and liver metastases. Accordingly, the patient was confirmed intraoperatively as having a GC-induced gastric perforation. The treatment strategy included primary gastric resection and repair surgery, which were primarily selected based on the presence of preoperative shock, the extent of peritonitis, neoplasm curability, and the patient’s comorbidities [25]. Primary gastric resection involves gastrectomy with radical lymphadenectomy and palliative gastrectomy. Repair surgery is usually performed to close the perforation using an omental patch. Following repair surgery, patients frequently undergo a secondary gastric resection or are administered chemotherapy for GC. In our case, gastrectomy with LN dissection was performed, because we had diagnosed a GC-induced perforation without peritoneal dissemination or distant metastases (except for a solitary liver metastasis) and the patient’s general condition was stable without comorbidities. Since the patient suffered from severe perioperative peritonitis, we anticipated that a high degree of peritoneal adhesion would arise from severe peritoneal inflammation. Therefore, we believed that if, during the initial operation, we had performed gastrectomy or repair surgery without lymphadenectomy, then it would have been difficult to perform surgery with lymphadenectomy at a later date. To the best of our knowledge, the only previous case of GHAC involving a gastric perforation has been reported in a pediatric patient [26]. Therefore, our case is the first involving an adult patient.

Currently, a standard treatment regimen for GHAC is lacking and treatments designed for GC are being administered. In instances where GHAC is accompanied by liver metastases, radical resection should be considered, provided the number of liver metastases is limited and liver resection is achievable [20, 27]. However, in a significant proportion of GHAC patients, early recurrence occurs, even when preventative LN dissection and radical resection have been performed [3]. In contrast, palliative surgery may be conducted in instances where distant metastases are present and radical resection is not achievable. In our case, since the liver metastasis was solitary, liver resection was performed and no metastases to the LNs or other organs were observed during the 11-week follow-up period. Unfortunately, however, LN metastases were detected shortly thereafter.

Although the cause is unknown, LN and liver metastases could easily develop given the extent of LN and venous invasion accompanying GHACs. GHACs also progress relatively rapidly. Therefore, GHAC is considered to be highly malignant [28]. Nagai et al. [5] reported that GHAC is associated with a poor prognosis compared to other forms of AFP-producing GC, which typically have a poorer prognosis than classical GC. Beak et al. [3] reported mean survival periods of 28.0 and 8.0 months for Stage I–III and Stage IV patients, respectively. Recently, Qu et al. [4] reviewed 95 GHAC cases from China and reported a 3-year survival rate of 7.4% and a median survival time of 10 months. The high malignancy of GHAC is associated with a high degree of tumor vascularization and rapid cell proliferation. Koide et al. [29] reported elevated levels of the Ki-67 labeling index and vascular endothelial growth factor expression in AFP-producing versus non-AFP-producing GC patients. Moreover, Inagawa et al. [8] demonstrated elevated levels of Ki-67 expression in GHAC tumors that was associated with tumor cell proliferation, relatively weak apoptosis, and rich neovascularization.

Treatment for GHAC patients with unresectable metastases, including S-1 monotherapy and paclitaxel/cisplatin-based, oxaliplatin/capecitabine-based, and fluoropyrimidine/platinum-based chemotherapy has been effective in some instances [3, 30]. However, to date, no antitumor drug has been established as the gold standard of treatment. In our case, shrinkage of the recurrent LN metastases and normalized serum AFP levels were observed following administration of S-1 monotherapy and a steady effect with respect to GHAC was achieved.

To improve the prognoses of patients with GHAC and AFP-producing GC, as well as, diagnostic accuracy and incorporation of radical resections, multimodality therapy (including supplementary chemotherapy) and the establishment of a standard chemotherapy regimen are required to address instances of recurrent tumors. Even when radical resections are performed, recurrences and additional metastases frequently occur. Therefore, closer monitoring is needed.

We describe a rare case of GHAC resulting in a spontaneous gastric perforation. In our patient, the GHAC was accompanied by multiple LN metastases and a solitary liver metastasis. Distal gastrectomy and lateral liver lobe resection were performed. However, the patient developed postoperative recurrence of LN metastasis. S-1 monotherapy was administered and complete response of the LN metastases was achieved.

GHACs progress rapidly and are characterized by a high frequency of LN and liver metastases. Therefore, GHACs are considered highly malignant tumors with poor prognoses. We anticipate that both clinical and basic research will continue to advance with the accumulation of future cases.

## Abbreviations

AFP: Alpha-fetoprotein
CT: Computed tomography
GC: Gastric cancer
GHAC: Gastric hepatoid adenocarcinoma
HCC: Hepatocellular carcinoma
LN: Lymph node
S-1: Tegafur/gimeracil/oteracil
SALL4: Sal-like protein 4
